# Concurrent ANCA-associated vasculitis and IgG4-related disease in a patient with fever of unknown origin and acute kidney injury: A case report

**DOI:** 10.1097/MD.0000000000041410

**Published:** 2025-01-31

**Authors:** Soo Jin Lee, Yujin Shin, Seung Ah Cha, Kyoung Min Kim, Kyung Pyo Kang

**Affiliations:** a Department of Internal Medicine, Jeonbuk National University Hospital, Jeonju, Korea; b Department of Internal Medicine, Research Institute of Clinical Medicine, Jeonbuk National University Medical School, Jeonju, Korea; c Department of Pathology, Jeonbuk National University Medical School, Jeonju, Korea.

**Keywords:** acute kidney injury, ANCA-associated vasculitis, fever of unknown origin, IgG4-related disease

## Abstract

**Rationale:**

It is often challenging to differentiate between IgG4-related disease (IgG4-RD) and antineutrophil cytoplasmic antibody-associated vasculitis (AAV) due to their similar clinical presentations. Recently, growing evidence has suggested a strong connection between AAV and IgG4-RD.

**Patient concerns:**

A 60-year-old woman was transferred to our hospital with fever and kidney dysfunction. Abdominal computed tomography revealed widespread infiltrative lesions in both kidneys.

**Diagnoses:**

Laboratory tests and subsequent renal biopsy confirmed both antineutrophil cytoplasmic antibody-associated vasculitis and IgG4-related disease.

**Interventions:**

We initiated plasmapheresis, oral cyclophosphamide, and high-dose glucocorticoids for treatment. Despite this, the patient’s condition worsened, requiring emergency hemodialysis.

**Outcomes:**

After 3 months of continued immunosuppressive treatment, renal function improved and hemodialysis was discontinued.

**Lessons:**

Our case showed an overlap of AAV and IgG4‐RD, which might support the hypothesis of an overlap syndrome of AAV and IgG4-RD. Clinicians should have a high index of suspicion when diagnosing fever of unknown origin, with the possibility of overlapping AAV and IgG4-RD.

## 1. Introduction

IgG4-related disease (IgG4-RD) is an immune-mediated fibroinflammatory condition that involves multiple systems such as the pancreas, biliary tree, salivary gland, kidney, mediastinum, and retroperitoneal spaces.^[1]^ It is characterized by dense lymphoplasmacytic infiltration rich in IgG4-positive plasma cells with the formation of mass-like or storiform fibrotic lesions and obliterative phlebitis surrounded by lymphocytes and plasma cell infiltration.^[2]^ Elevated numbers of IgG4+ plasma cells in tissue and serum IgG4 levels may be helpful in the diagnosis of IgG4-related diseases, but they are not specific diagnostic markers.^[1,2]^ Antineutrophil cytoplasmic antibody (ANCA)-associated vasculitis (AAV) are multi-system autoimmune diseases involving small vessels, characterized by pauci-immune necrotizing vasculitis, which can include crescentic glomerulonephritis.^[3]^ Substantial overlap between AAV and IgG4-RD exists with respect to organ involvement and histopathological features.^[4]^ Clinically, orbital mass, tubulointerstitial nephritis, renal mass, thyroiditis, mediastinal fibrosis, and periaortitis may be observed in IgG4-RD and AAV.^[5]^ In this report, we describe a patient presenting with fever of unknown origin (FUO) who was diagnosed with overlapping AAV and IgG4-RD by renal biopsy.

## 2. Ethical approval

Ethical approval was obtained from the Jeonbuk National University Hospital Institutional Review Board (CUH 2022-02-001). Written informed consent for publication was obtained from the patient.

## 3. Case

**Figure 1.**
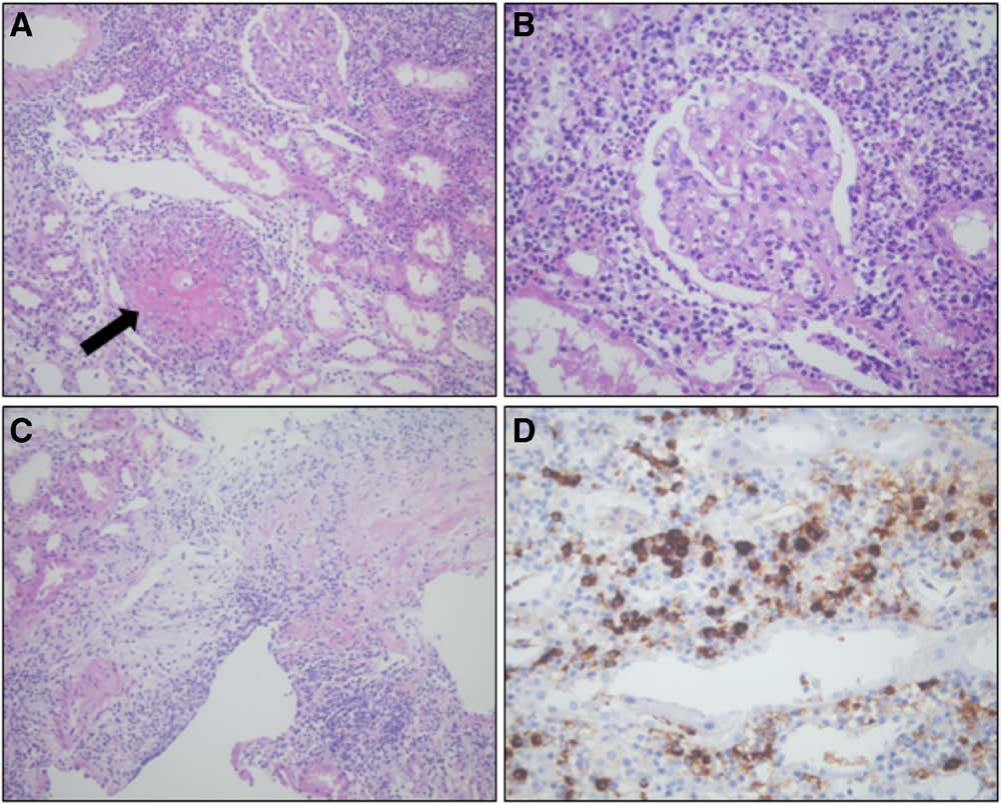
Histologic feature of renal biopsy. (A) Low power-view showing diffuse inflammatory infiltration in interstitium and necrotizing vasculitis (arrow; periodic acid–Schiff [PAS] stain, original magnification: ×200). (B) Neutrophil dominant inflammatory infiltration is noted in interstitium and also in glomerular capillary lumina (PAS stain, original magnification: ×400). (C) Other area showing interstitial fibrosis associated with diffuse lymphoplasmacytic infiltration (PAS stain, original magnification: ×200). (D) Immunohistochemical staining for IgG4 showing markedly increased IgG4-positive plasma cells (>30 plasma cells/HPF) (original magnification: ×400).

**Figure 2.**
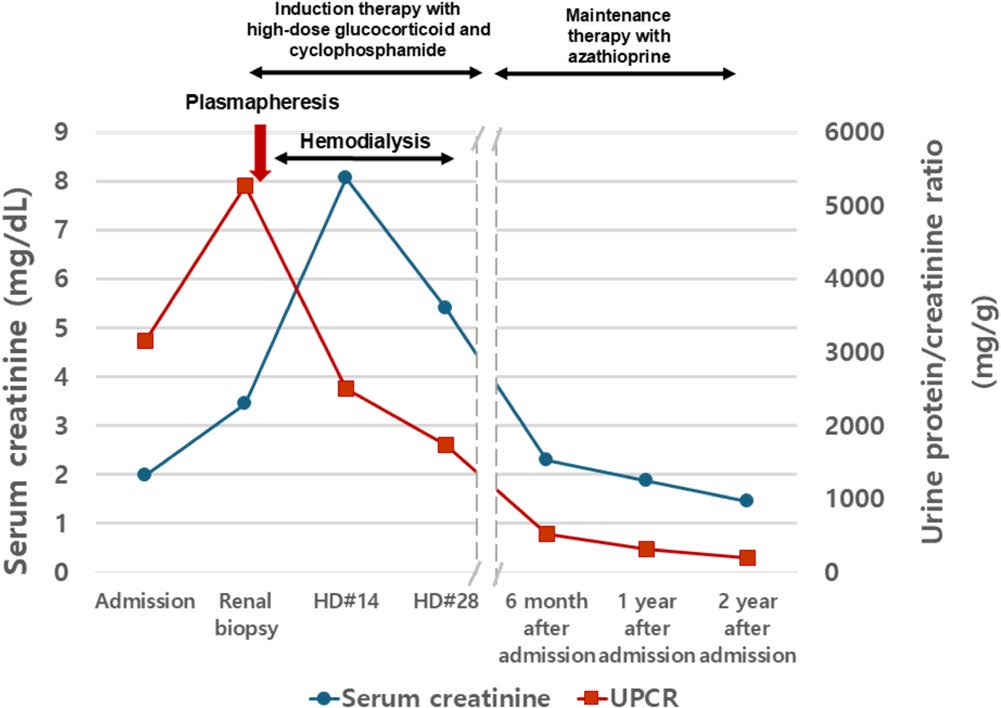
The clinical course after renal biopsy.

A 60-year-old previously healthy woman was referred to our hospital for fever and renal dysfunction. Physical examination revealed costovertebral angle tenderness and 1 + pretibial pitting edema. Initial laboratory results were as follows: white blood cell count, 15,820/mm^3^; hemoglobin level, 9.3 g/dL, platelet count, 442,000/mm^3^; high-sensitivity C-reactive protein, 202.54 mg/L, and serum creatinine level, 1.99 mg/dL. Urinalysis revealed 3 + proteinuria with microscopic hematuria. The urine protein/creatinine ratio was 3159 mg/g creatinine. The antinuclear antibody test result was negative. The antimyeloperoxidiase (MPO)-ANCA test results were positive (214 U/mL). However, antiglomerular basement membrane (GBM) and antiproteinase 3 (PR3)-ANCA results were negative. In addition, the serum IgG/IgG4 levels were elevated at 2158.9 mg/dL (reference range, 700–1600 mg/dL) and 374 mg/dL (reference range, ≤135mg/dL). The serum protein electrophoresis and urine protein electrophoresis test showed nonspecific findings. An abdominal computed tomography scan was performed before transfer to our hospital, showing diffuse infiltrative lesions in both kidneys (Supplemental Digital Content, http://links.lww.com/MD/O330, which shows abdominal computed tomography scan result). Blood and urine cultures showed no growth on day 7. The fever persisted, and renal function deteriorated to an increasing creatinine level of 2.52 mg/dL. A renal biopsy was performed. Renal biopsy revealed glomerular thickening with lymphocytic and neutrophilic infiltration, and tubulointerstitial areas had diffuse neutrophilic and lymphoplasmacytic infiltration and fibrinoid necrotizing arteritis with neutrophilic infiltration. Because of the elevated serum IgG4 levels, we performed immunohistochemical staining for IgG4. There were >30 IgG4-positive plasma cells/high power field (HPF). These findings were consistent with ANCA-associated glomerulonephritis, mixed class, and IgG4-RD (Fig. 1). We initiated plasmapheresis and oral cyclophosphamide 75 mg with high-dose glucocorticoid (1 mg/kg) treatment. Despite immunosuppressive treatment, the patient’s condition deteriorated, and shortness of breath associated with pulmonary edema and pleural effusion developed. Therefore, emergency hemodialysis was initiated. After 3 months of high-dose prednisolone treatment with oral cyclophosphamide, renal function recovered to a serum creatinine of 3.98 mg/dL, and hemodialysis was discontinued. We switched oral glucocorticoid and cyclophosphamide to azathioprine for maintenance therapy according to Korean National Health Insurance Service guideline. The patient had stable renal function with a creatinine level of 1.45 mg/dL after 2 years of initial diagnosis (Fig. 2).

## 4. Discussion

AAV and IgG4-RD are immune-related disorders characterized by the formation of autoimmune antibodies, such as ANCA and IgG4 antibodies, in their pathogenesis. Here, we present a patient with AAV combined with IgG4-RD that was successfully treated with timely renal biopsy and immunosuppressive agents. Even though AAV and IgG4-RD have different etiologies, they can overlap.

However, there are several controversies regarding the overlap of AAV and IgG4-RD. Previously, to differentiate IgG4-RD from similar diseases, the initial inclusion and exclusion criteria were established, with ANCA positivity being one of the exclusion criteria. However, recent findings indicate that IgG4-RD and AAV can co-occur.^[5]^ They share similarities in clinical, serological, radiological, and histopathological features, making it difficult to distinguish and complicating the differential diagnosis.^[6]^ Recent reports have indicated the potential for overlap syndrome involving IgG4-RD and AAV, suggesting a possible connection between these conditions. The connection between the pathological roles of ANCA and IgG4 in causing damage in both diseases remains unclear. Some evidence highlights the involvement of T follicular helper cells, which are elevated under both conditions.^[5]^ Others are related to chronic inflammatory processes and accumulation of self-reactive B cells that produce various autoantibodies.^[7]^ Activation of T-regulatory cells and T-helper2 response is responsible for elevated IgG4 levels in IgG4-RD.^[8]^ ANCA predominantly belongs to the IgG1 and IgG4 subclasses, which can activate neutrophils and lead to AAV.^[9]^

This case highlights the presence of both conditions in the symptoms of FUO: IgG4-related tubulointerstitial nephritis, the most common manifestation of IgG4-related kidney disease, and ANCA-associated vasculitis. A biopsy is essential for the diagnosis of AAV and IgG4-RD. However, in some cases, a biopsy cannot be performed because of the patient’s preference or localization of the involved tissue, such as aortitis. Therefore, clinical features and supportive imaging or laboratory data may provide important diagnostic clues.

In conclusion, we report a patient with AAV combined with IgG4-RD, who was successfully treated with timely renal biopsy and immunosuppressive treatment. Clinicians should have a high index of suspicion when investigating FUO, with the possibility of underlying autoimmune disease, including an overlap picture as in this patient.

## Author contributions

**Data curation:** Soo Jin Lee, Yujin Shin, Seung Ah Cha.

**Writing – original draft:** Soo Jin Lee, Seung Ah Cha.

**Formal analysis:** Kyung Min Kim, Kyung Pyo Kang.

**Validation:** Kyung Min Kim, Kyung Pyo Kang.

**Conceptualization:** Kyung Pyo Kang.

**Funding acquisition:** Kyung Pyo Kang.

**Supervision:** Kyung Pyo Kang.

**Writing – review & editing:** Kyung Pyo Kang.

## Supplementary Material


