# Supplementary figures and images for: Concurrent ANCA-associated vasculitis and IgG4-related disease in a patient with fever of unknown origin and acute kidney injury: A case report
Source: Medicine (Baltimore). 2025 Jan 31;104(5):e41410. doi: 10.1097/MD.0000000000041410 (PMC11789897; doi:10.1097/MD.0000000000041410)

## Slide 1
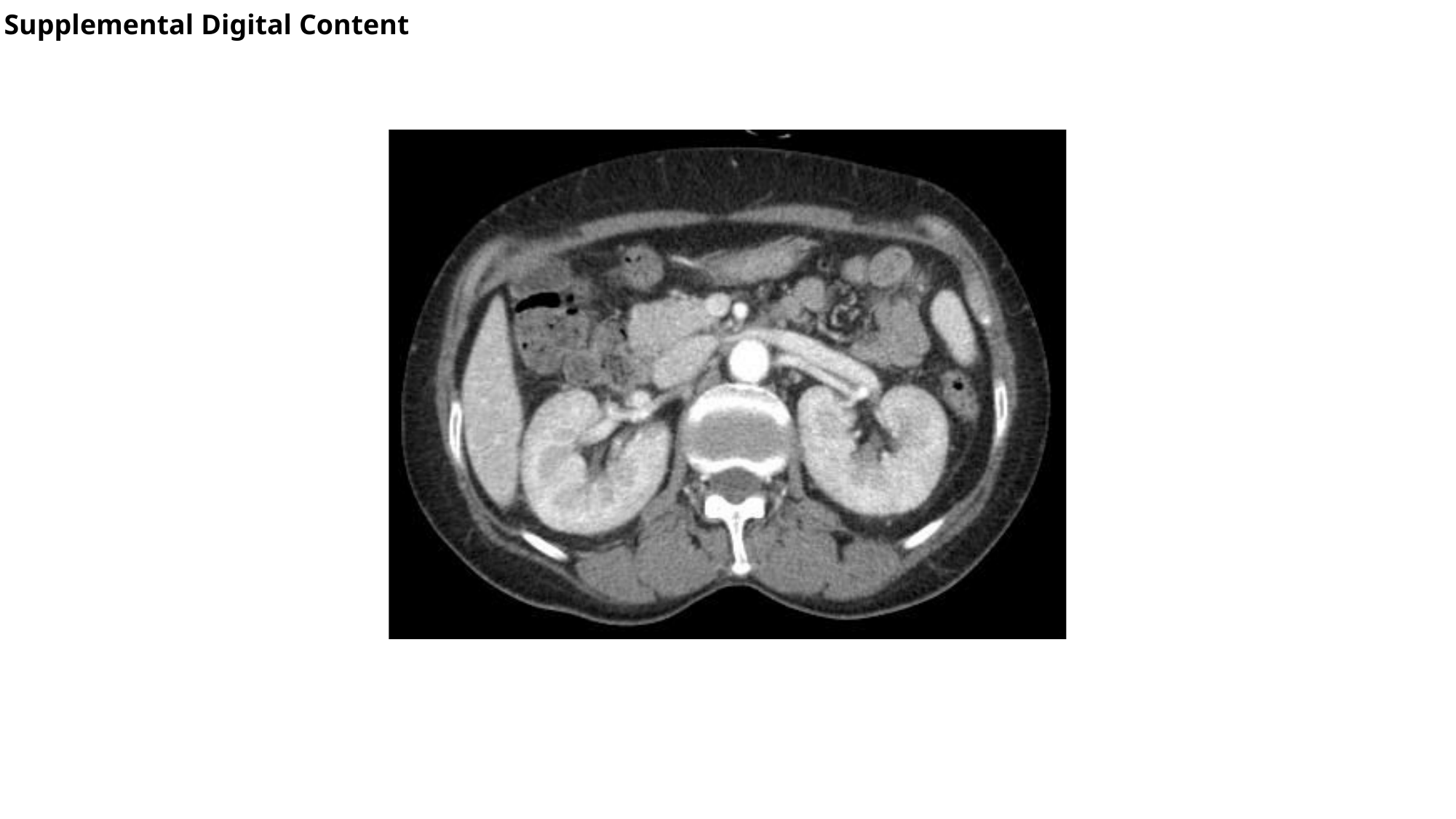

Supplemental Digital Content

Supplement: Supplementary file 1 [file medi-104-e41410-s001.pptx]
